# Realistic High-Resolution Body Computed Tomography Image Synthesis by Using Progressive Growing Generative Adversarial Network: Visual Turing Test

**DOI:** 10.2196/23328

**Published:** 2021-03-17

**Authors:** Ho Young Park, Hyun-Jin Bae, Gil-Sun Hong, Minjee Kim, JiHye Yun, Sungwon Park, Won Jung Chung, NamKug Kim

**Affiliations:** 1 Department of Radiology and Research Institute of Radiology, University of Ulsan College of Medicine & Asan Medical Center Seoul Republic of Korea; 2 Department of Medicine, University of Ulsan College of Medicine & Asan Medical Center Seoul Republic of Korea; 3 Department of Biomedical Engineering, Asan Medical Institute of Convergence Science and Technology, Asan Medical Center, University of Ulsan College of Medicine, Seoul, Republic of Korea Seoul Republic of Korea; 4 Department of Health Screening and Promotion Center, University of Ulsan College of Medicine & Asan Medical Center Seoul Republic of Korea; 5 Department of Convergence Medicine, University of Ulsan College of Medicine & Asan Medical Center Seoul Republic of Korea

**Keywords:** generative adversarial network, unsupervised deep learning, computed tomography, synthetic body images, visual Turing test

## Abstract

**Background:**

Generative adversarial network (GAN)–based synthetic images can be viable solutions to current supervised deep learning challenges. However, generating highly realistic images is a prerequisite for these approaches.

**Objective:**

The aim of this study was to investigate and validate the unsupervised synthesis of highly realistic body computed tomography (CT) images by using a progressive growing GAN (PGGAN) trained to learn the probability distribution of normal data.

**Methods:**

We trained the PGGAN by using 11,755 body CT scans. Ten radiologists (4 radiologists with <5 years of experience [Group I], 4 radiologists with 5-10 years of experience [Group II], and 2 radiologists with >10 years of experience [Group III]) evaluated the results in a binary approach by using an independent validation set of 300 images (150 real and 150 synthetic) to judge the authenticity of each image.

**Results:**

The mean accuracy of the 10 readers in the entire image set was higher than random guessing (1781/3000, 59.4% vs 1500/3000, 50.0%, respectively; *P*<.001). However, in terms of identifying synthetic images as fake, there was no significant difference in the specificity between the visual Turing test and random guessing (779/1500, 51.9% vs 750/1500, 50.0%, respectively; *P*=.29). The accuracy between the 3 reader groups with different experience levels was not significantly different (Group I, 696/1200, 58.0%; Group II, 726/1200, 60.5%; and Group III, 359/600, 59.8%; *P*=.36). Interreader agreements were poor (κ=0.11) for the entire image set. In subgroup analysis, the discrepancies between real and synthetic CT images occurred mainly in the thoracoabdominal junction and in the anatomical details.

**Conclusions:**

The GAN can synthesize highly realistic high-resolution body CT images that are indistinguishable from real images; however, it has limitations in generating body images of the thoracoabdominal junction and lacks accuracy in the anatomical details.

## Introduction

Generative adversarial networks (GANs) is a recent innovative technology that generates artificial but realistic-looking images. Despite the negative views regarding the use of synthetic images in the medical field, GANs have been spotlighted in radiological research because of their undeniable advantages [1]. The use of diagnostic radiological images in the public domain always raises the problem of protecting patients’ privacy [2-5]. This has been a great challenge to researchers in the field of deep learning. GANs may provide a solution to these privacy concerns. Moreover, GANs are powerful nonsupervised training methods. The traditional supervised learning methods have been challenged by a lack of high-quality training data labelled by experts. Building these data requires considerable time input from experts and leads to correspondingly high costs [6]. This problem has not yet been resolved despite several collaborative efforts to build large open access data sets [7]. Most radiological tasks using GANs include the generation of synthetic images for augmenting training images [8-11], translation between different radiological modalities [12-16], image reconstruction and denoising [17-20], and data segmentation [21-24].

The more recent noteworthy task using GANs is anomaly detection. Unlike other tasks using GANs, detecting abnormalities is based on learning the probability distribution of normal training data. Image data outside this distribution are considered as abnormal. Schlegl et al [25] demonstrated GAN-based anomaly detection in optical coherence tomography images. They trained GAN with normal data in an unsupervised approach and proposed an anomaly scoring scheme. Alex et al [26] showed that GAN can detect brain lesions on magnetic resonance images. This approach has attracted many radiologists for several reasons; the most critical is that this approach can achieve a broader clinical application than the current supervised deep learning–based diagnostic models. In daily clinical practice, diagnostic images are clinically acquired for patients with a variety of diseases. Therefore, before applying the supervised deep learning model, it is necessary to select suspected disease cases with disease categories similar to those of a training data set. For example, in the emergency department, a deep learning model trained by data from patients with acute appendicitis could hardly be applied to patients with different abdominal pathologies.

For this approach, we think that generating highly realistic images is a prerequisite. Previous studies [25,26] trained a GAN model with small patches (64×64 pixels), which are randomly extracted from original images. The trained model could only generate small patches and did not learn the semantics of the whole images. Hence, the GAN model may generate artificial features, which can lead to large errors in anomaly detection tasks. In addition, there are various kinds of small and subtle lesions in the actual clinical setting. Therefore, the previous low-resolution GAN approaches could not be used for this application. In this study, we trained GAN with whole-body computed tomography (CT) images (512×512 pixels); therefore, the model learned the semantics of the images. This may lead to robust performances in anomaly detection in CT images. Due to the aforementioned reasons, we have attempted to build large data sets of normal medical images to develop GAN-based diagnostic models for clinical application. As a preliminary study, we investigated and validated the unsupervised synthesis of highly realistic body CT images by using GAN by learning the probability distribution of normal training data.

## Methods

### Ethical Approval

This retrospective study was conducted according to the principles of the Declaration of Helsinki and was performed in accordance with current scientific guidelines. This study protocol was approved by the Institutional Review Board Committee of the Asan Medical Center (No. 2019-0486). The requirement for informed patient consent was waived.

### Data Collection for Training

We retrospectively reviewed electronic medical records of patients who underwent chest CT or abdominopelvic CT (AP-CT) in the Health Screening and Promotion Center of Asan Medical Center between January 2013 and December 2017. We identified 139,390 patients. Their radiologic reports were then reviewed using the radiologic diagnostic codes “Code 0” or “Code B0,” which indicated normal CT in our institution’s disease classification system, and 17,854 patients with normal chest CT or normal AP-CT were identified. One board-certified radiologist (GSH) reviewed the radiological reports of the 17,854 patients and excluded 3650 cases with incidental benign lesions (eg, hepatic cysts, renal cysts, thyroid nodules) detected on body CT images. Benign lesions were defined as positive incidental findings on CT images, which did not require medical or surgical intervention. Our final study group included CT images showing anatomical variations (eg, right aortic arch, double inferior vena cava) and senile changes (eg, atherosclerotic calcification without clinical significance). Of the potentially suitable 14,204 cases, 2449 CT data sets were not available for automatic download using the inhouse system of our institution. Finally, this study included 11,755 body CT scans (473,833 axial slices) for training the GAN, comprising 5000 contrast-enhanced chest CT scans (172,249 axial slices) and 6755 AP-CT scans (301,584 axial slices, comprising 132,880 slices of contrast-enhanced AP-CT and 168,704 slices of contrast-enhanced low-dose AP-CT images).

### Training PGGAN to Generate Body CT Images

A progressive growing GAN (PGGAN) was used to generate high-resolution (512×512 pixels) synthetic body CT images. Unlike PGGAN, previous GAN models such as deep convolutional GANs were able to generate relatively low-resolution (256×256 pixels) synthetic images [27]. However, PGGANs have demonstrated that high-resolution images (1024×1024 pixels) can be generated by applying progressive growing techniques [28]. Because CT images are acquired in high resolutions (512×512 pixels), PGGAN could be the GAN model that can train with whole CT images in full resolution. Consequently, the GAN model can preserve their semantics in the original resolution of CT images. While StyleGAN also demonstrates realistic synthetic images with the style feature [29], we chose the PGGAN model for training because of its simple yet powerful performance. In addition, we did not consider BigGAN because it is a conditional model [30]. To train the PGGAN with body CT images, the original 12-bit grayscale CT images were converted into 8-bit grayscale potable network graphics images with 3 different windowing settings: (1) a lung setting (window width 1500, window level 600), (2) a mediastinal setting (window width 450, window level 50) for chest CT images, and (3) a multiorgan setting (window width 350, window level 40) for AP-CT images. Images from each group with different windowing settings were used to train a PGGAN separately.

A publicly available official implementation of PGGAN using Tensorflow in Python was used [31]. While the sizes of the training images progressively grew from 4×4 to 512×512 (ie, 2^n^×2^n^, where the integer n increases from 2 to 8), the batch sizes decreased from 512 to 16, respectively. The learning rate was fixed at 0.001 while training. We carefully monitored the training process (ie, training losses and generated images) with TensorBoard and intermediated image generation to determine whether the PGGAN was properly trained. The PGGAN training was completed after the network had evaluated around 20 million body CT images. The training took ~12.5 days with 2 NVIDIA Titan RTX graphic processing units for each group with different windowing settings (ie, total training for ~37.5 days).

### Visual Turing Test to Assess the Realistic Nature of Synthetic CT Images

**Figure 1:**
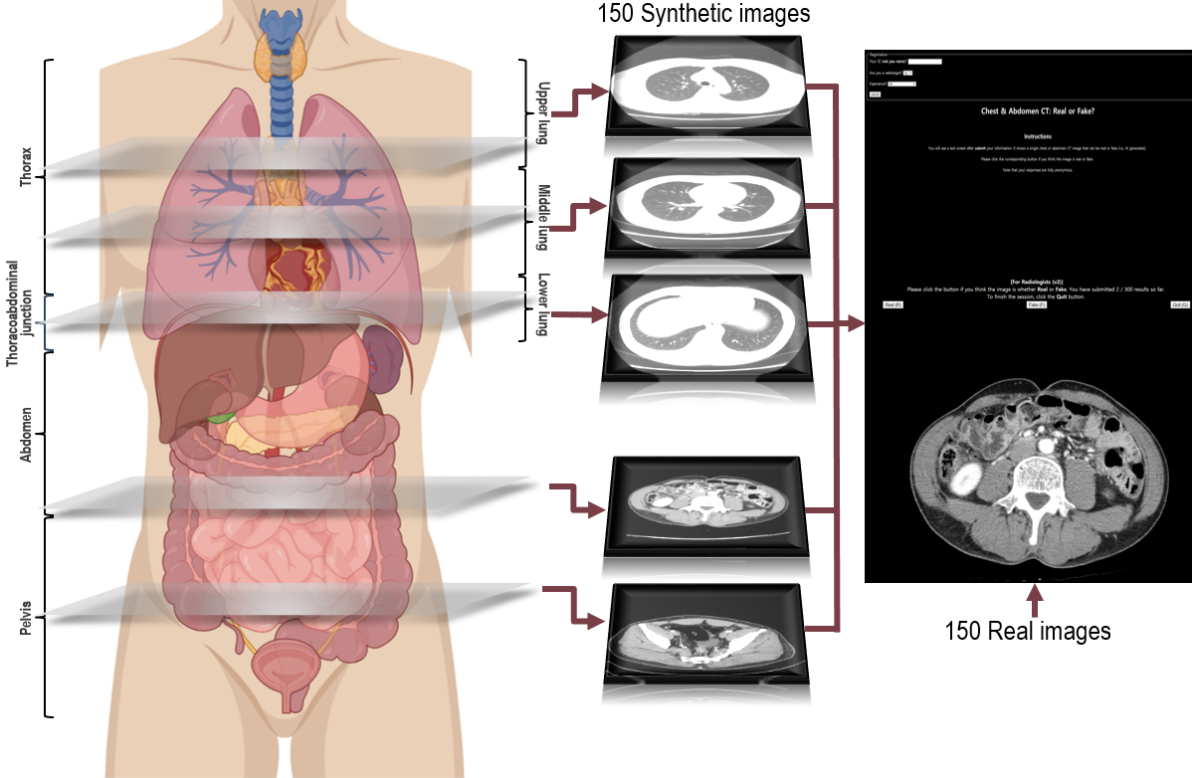
Graphical illustration of the method used to estimate the realism of the synthetic body computed tomography images. The validation set consisted of 150 synthetic and 150 real images. Synthetic images generated by the progressive growing generative adversarial network model and real images were randomly mixed and displayed on the website. Ten readers independently determined whether each image was real or artificial.

Figure 1 summarizes the study design for the visual assessment performed using an image Turing test. The validation set consisted of 300 axial body CT images (150 synthetic images and 150 real images). The 150 synthetic images comprised 50 chest CT-lung window (chest-L), 50 chest CT-mediastinal window (chest-M), and 50 AP-CT images. The validation set consisted of 7 subgroups based on the anatomical structure: 50 chest-L images were divided into upper lung, middle lung, and lower lung groups; and 50 chest-M and 50 AP-CT images were divided into thorax, thoracoabdominal junction, abdomen, and pelvis groups. To avoid any selection bias, all synthetic images in the validation set were automatically generated by the PGGAN model and were not individually selected by the researchers. For the real images, 50 CT images of each anatomical subgroup (ie, chest-L, chest-M, and AP-CT) were randomly selected from 50 normal whole-body CT scans (performed at the emergency department of Asan Medical Center) by 1 co-researcher (JHY) who did not otherwise participate in the realism assessment study. A website (validct.esy.es) was created to upload the validation set with 300 axial images posted and displayed in a random manner. Ten radiologists (4 radiologists with <5 years of experience [Group I], 4 radiologists with 5-10 years of experience [Group II], and 2 radiologists with >10 years of experience [Group III]) independently evaluated each of the 300 images slice-by-slice and decided whether each CT image was real or artificial by visual analysis with no time limit. To investigate the features of the images with obviously artificial appearance, we defined obviously artificial images as synthetic images that were identified as artificial by a majority of readers. Two radiologists (HYP and GSH) then visually reviewed these obviously artificial images. To determine whether the radiologists could learn to distinguish real from synthetic images, we performed an additional Turing test (postlearning visual Turing test). First, 2 board-certified radiologists (Group III) were educated in the obviously artificial findings in the synthetic images (not included in the test set). Then, 2 readers independently decided whether each of the 300 CT images were real or artificial by visual analysis. For accurate comparison of the results, the same test set as the index visual Turing test was used.

### Statistical Analyses

The mean accuracy, sensitivity, and specificity of the 10 readers were calculated. The generalized estimating equations method was used to test whether the ratio of mean accuracy and random guessing was 1. The generalized estimating equations were used to compare the accuracy, sensitivity, and specificity across the reader groups with different experience levels (Group I, Group II, and Group III) and across the anatomical subgroups. To compare the diagnostic performance among subgroups, chest-L was classified into 3 image subgroups (upper, middle, and lower lung), and chest-M and AP-CT images were grouped into 4 image subgroups (thorax, thoracoabdominal junction, abdomen, and pelvis) on the basis of anatomical structures by visual inspection. The anatomical landmarks used in subgrouping of CT-L were as follows: (1) upper lung: apex to upper border of tracheal bifurcation; (2) middle lung: upper border of tracheal bifurcation to upper border of diaphragm; and (3) lower lung: upper border of diaphragm to lower border of diaphragm. The anatomical landmarks used in the subgroups of CT-M and AP-CT were as follows: (1) thorax: apex to upper border of diaphragm; (2) thoracoabdominal junction: upper border of diaphragm to lower border of diaphragm; (3) abdomen: lower border of diaphragm to upper border of iliac crest; and (4) pelvis: below the upper border of iliac crest. Chest-M and AP-CT images were combined for the subgroup classification because these images included the “soft tissue setting” used for the whole body. Figure 1 shows the subgroup classification according to the anatomical level. The significance level was corrected for multiple comparisons using the Bonferroni correction. Interreader agreement was evaluated using Fleiss kappa. To identify obviously artificial images, a histogram analysis was used to display the distribution of the number of correct answers from the 10 readers (ie, identification of synthetic images as artificial) and the number of artificial images. The cut-off values (ie, percentage of readers with correct answers) were set where dramatic changes in the histogram distribution was observed. When a cut-off ≥70% was used for chest-L and ≥80% for chest-M and AP-CT images, 1 subgroup (ie, upper lung for chest-L and thoracoabdominal junction for chest-M and AP-CT images) had the highest number of readers with correct answers. In the postlearning visual Turing test, the mean accuracy, sensitivity, and specificity of the 2 readers were calculated. SPSS software (version 23, IBM Corp) and R version 3.5.3 (R Foundation for Statistical Computing) were used for the statistical analyses with the significance level set at *P*<.05.

## Results

### Results of the Visual Turing Test

**Figure 2:**
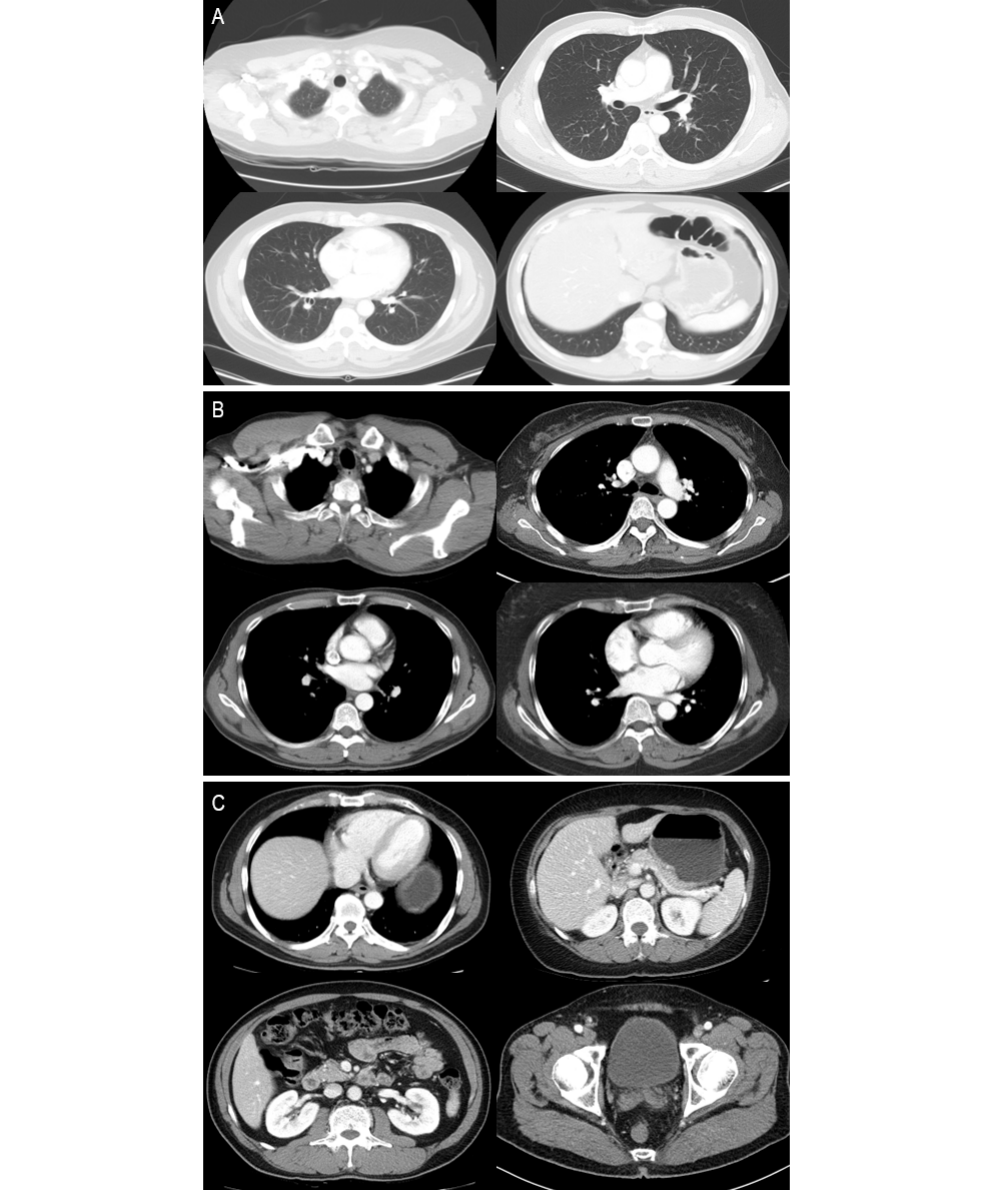
Synthetic high-resolution body computed tomography images. A. Chest computed tomography images-lung window. B. Chest computed tomography images-mediastinal window. C. Abdominopelvic computed tomography images.

Table 1 summarizes the results of the realism assessment of all images by the 10 readers. The mean accuracy of the 10 readers in the entire image set was higher than the random guessing (1781/3000, 59.4% vs 1500/3000, 50.0%, respectively; *P*<.001). However, in terms of identifying synthetic images as fake, there was no significant difference in the specificity between the visual Turing test and random guessing (779/1500, 51.9% vs 750/1500, 50.0%, respectively; *P*=.29). There was no significant difference in the accuracy between the 3 reader groups with different experience levels (Group I, 696/1200, 58.0%; Group II, 726/1200, 60.5%; and Group III, 359/600, 59.8%; *P*=.36). In the detection of synthetic images, Group III showed a significantly lower specificity than Group II (*P*=.01) but did not show a significant difference from Group I (*P*=.30). Multimedia Appendix 4 summarizes the results of the subgroup analysis of the realism assessment according to the anatomical region. There were no significant differences in the accuracy between the 3 CT groups (chest-L, 595/1000, 59.5%; chest-M, 615/1000, 61.5%; and AP-CT, 571/1000, 57.1%; *P*=.33). In addition, there was no significant difference in the accuracy between the upper, middle, and lower lung groups of the chest-L images (upper lung, 227/370, 61.4%; middle lung, 190/290, 65.5%; and lower lung, 136/240, 56.7%, *P*=.36). The thoracoabdominal junction showed a significantly higher accuracy (208/280, 74.3% vs 194/370, 52.4% to 361/600, 60.2%; *P*=.004) and specificity (154/200, 77.0% vs 93/220, 42.3% to 149/250, 59.6%; *P*<.001) compared with the other subgroups. Examples of the multilevel random generation of synthetic chest CT and AP-CT images by the PGGAN are shown in Figure 2 and in Multimedia Appendix 1, Multimedia Appendix 2, and Multimedia Appendix 3.

**Table 1:** Assessment of the realism of all images by the 10 readers.

Groups, readers (R)		Accuracy (%)^a^	Sensitivity (%)^b^	Specificity (%)^c^
**Group I^d^**				
	R01	56.7	67.3	46.0
	R05	48.3	53.3	43.3
	R09	61.0	70.7	51.3
	R10	66.0	70.7	61.3
**Group II^e^**				
	R02	43.7	50.0	37.3
	R06	73.0	68.7	77.3
	R07	61.3	65.3	57.3
	R08	64.0	77.3	50.7
**Group III^f^**				
	R03	65.3	86.0	44.7
	R04	54.3	58.7	50.0

^a^Mean (95% CI) accuracy: 59.4 (56.9-61.8), *P*=.36. *P* value was determined by generalized estimating equations.

^b^Mean (95% CI) sensitivity: 66.8 (63.9-69.5), *P*=.04.

^c^Mean (95% CI) specificity: 51.9 (48.4-55.5), *P*=.02.

^d^Group I: radiologists with <5 years of experience. Mean (95% CI) accuracy 58.0 (55.0-61.0), sensitivity 65.5 (61.4-69.4), and specificity 50.5 (46.3-54.7).

^e^Group II: radiologists with 5-10 years of experience. Mean (95% CI) accuracy 60.5 (57.6-63.4), sensitivity 65.3 (61.4-69.0), and specificity 55.7 (51.4-59.9).

^f^Group III: radiologists with >10 years of experience. Mean (95% CI) accuracy 59.8 (55.5-64.1), sensitivity 72.3 (67.0-77.1), and specificity 47.3 (41.1-53.7).

In the postlearning visual Turing test, the mean accuracy, sensitivity, and specificity of the 2 radiologists were 67.3%, 72.7%, and 62.0%, respectively. Compared with the results of the index visual Turing test, the accuracy was increased by 7.5% and the specificity was increased by 10.1% in the postlearning visual Turing test.

### Interreader Agreement for Synthetic and Real Images

Interreader agreement was poor for the entire image set (κ=0.11) and for the 3 CT subsets (chest-L, chest-M, and AP-CT; κ=0.04-0.13). Interreader agreement was higher for the thoracoabdominal junction subset than for the other anatomical regions (κ=0.31 vs 0.03-0.14) (Table 2).

**Table 2:** Interreader agreement of the 10 readers with respect to the imaging subgroups.

Image type, subsets			Kappa values		95% CI
Entire image set			0.11		0.09 to 0.13
**Image subsets**					
	Chest-L^a^	0.04		0.01 to 0.07	
	Chest-M^b^	0.13		0.10 to 0.15	
	AP-CT^c^	0.11		0.08 to 0.14	
**Chest-L**					
	Upper lung	0.04		–0.01 to 0.09	
	Middle lung	0.01		–0.04 to 0.07	
	Lower lung	0.06		0.00 to 0.12	
**Chest-M and AP-CT**					
	Thorax	0.03		–0.01 to 0.06	
	Thoracoabdominal junction	0.31		0.25 to 0.36	
	Abdomen	0.14		0.10 to 0.18	
	Pelvis	0.03		–0.02 to 0.08	

^a^Chest-L: chest computed tomography images-lung window.

^b^Chest-M: chest computed tomography images-mediastinal window.

^c^AP-CT: abdominopelvic computed tomography images.

### Analysis of the Features of Obviously Artificial Images

**Figure 3:**
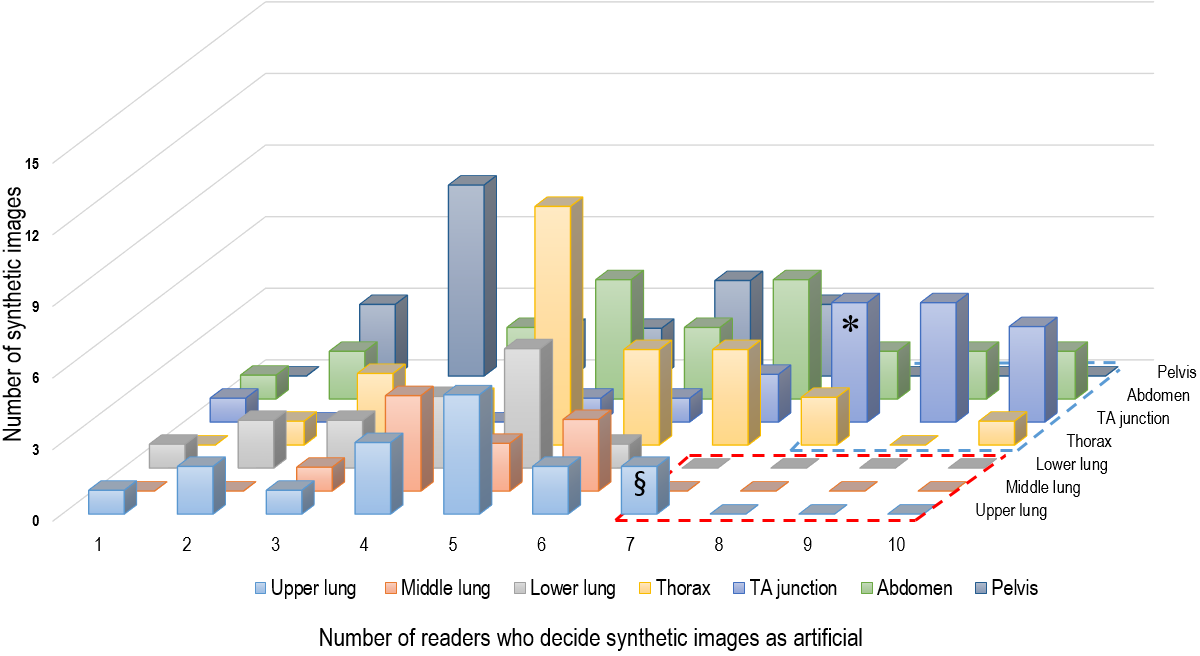
Histogram analysis of the correct answers for the 150 synthetic images (accurate identification of the artificial images) by the 10 readers. A. When a cut-off for the percentage of readers with correct answers was set at ≥70% for the chest computed tomography-lung window group, only 1 subgroup (upper lung) remained (§). B. When a cut-off level for the percentage of readers with correct answers was set at ≥80% for the chest computed tomography-mediastinal window and abdominopelvic computed tomography groups, the thoracoabdominal (TA) junction group (*) showed dominance over the other subgroups.

**Figure 4:**
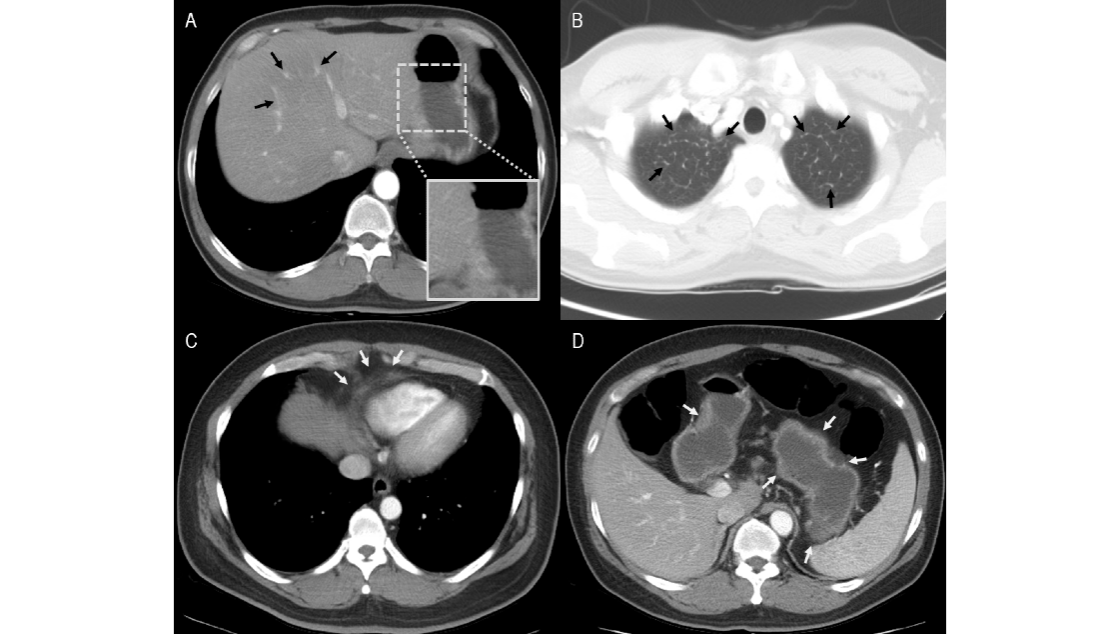
Obviously artificial body computed tomography images. A. Ill-defined margins and abnormal courses of intrahepatic vessels (arrows) in the liver. Note curvilinear structures (dotted rectangle) at the liver and stomach. B. Accentuated vascular markings in both upper lung apices (arrows). C. Abnormal infiltration in the pericardial fat (arrows). D. Irregular contours of the stomach body and antrum with blurred margins (arrows).

Figure 3 shows that the majority of readers characterized the synthetic images as artificial predominantly at the thoracoabdominal junction of the chest-M and AP-CT, followed by the upper lung of the chest-L. Using a histogram analysis, 24 of the 150 synthetic images (22 images of the chest-M and AP-CT groups and 2 images of the upper lung) were selected and reviewed by 2 radiologists to identify the features indicating that the images were artificial. Table 3 details the artificial features indicative of synthetic CT images. A total of 34 artificial features were found in the 24 synthetic images, the most common being vascular structures (24/34, 71%), followed by movable organs (ie, stomach, heart, small bowel, and mediastinal fat around the heart, 8/34, 24%). Among the vascular structures, intrahepatic vessels (ie, portal and hepatic veins) most frequently had abnormal configurations, directions, or diameters (Figure 4). In case of the movable organs, an abnormal organ contour was the main feature indicative of an artificially generated image (Figure 4C and Figure 4D).

**Table 3:** Details of the obviously artificial body computed tomography images.

Configuration, artificial features			Images (n)
**Abnormal vascular configuration^a^**			
	Hepatic vessel (portal vein and hepatic vein)	13	
	Gastric vessel	3	
	Mesenteric vessel	2	
	Pulmonary vessel	2	
	Others (peripancreatic, coronary, rectal, axillary vessel)	4	
**Abnormal contour or structure^b^**			
	Stomach	3	
	Pancreas	2	
	Heart	2	
	Mediastinal fat around the heart	2	
	Small bowel	1	

^a^Ill-defined vascular margin, bizarre vascular course, or abnormal vascular diameter.

^b^Blurred margin of the organ, or bizarre structure of the soft tissue.

## Discussion

### Principal Findings

We showed that the GAN-based synthetic whole-body CT images have comparable image fidelity to real images. For this, our study validated the synthetic images by multiple radiology experts because the visual Turing test could be greatly influenced by the reader’s level of expertise [10,32,33]. There was no significant difference in the accuracy between the reader groups. In addition, the interreader agreement was poor for the distinction between real and synthetic images. These results imply that a validation test was properly performed with mitigation of the impact of the reader’s level of expertise. However, there was quite a significant disparity between sensitivity (66.8%) and specificity (51.9%). We presume that this is mainly due to factors affecting reader performance test. First, all readers had at least some exposure to real body CT images in clinical practice. In addition, the real images in the validation data set consisted of relatively uniform CT images because they were acquired using a similar CT machine with similar acquisition parameters. These factors affect the readers’ confidence and decisions to identify real images, resulting in high sensitivity. This is supported by the fact that the sensitivity proposed here reached 72.3% in Group III (radiologists with long-term exposure to real CT images in our institution). In contrast, some obviously artificial features (eg, the ill-defined margin of the heart) in synthetic images are similar to the motion artifacts or noises in real images. This can cause reader confusion, resulting in lower specificity. In addition, the mean accuracy (59.4%) was higher than random guessing (50%); however, it is believed that the high sensitivity contributed significantly to this result. Therefore, in terms of identifying synthetic images as fake, the readers’ performance was not much better than random guessing. For robust validation, using real CT images from other medical institutions (not experienced by the readers) in the validation set could be needed. Despite this limitation, our data suggest that the synthetic images are highly realistic and indistinguishable from real CT images.

One critical finding of this study was that the discrepancies between real and synthetic CT images occur mainly in the thoracoabdominal junction and in anatomical details. The thoracoabdominal junction is the most prone to motion artifacts due to respiratory movement. In addition, it has a complex anatomical structure due to multiple organs in small spaces [34]. These features of the thoracoabdominal junction might have contributed to the identification of unrealistic synthetic body images. This phenomenon in the areas with complex structures has been shown in other image syntheses using GANs [27,28]. It is worth noting that this study showed that GAN achieved highly realistic images for gross anatomy and not for detailed anatomical structures. The most common obviously artificial features in synthetic images were bizarre configurations and directions of small-to-medium vessels. This is probably due to the lack of the interslice shape continuity caused by the 2D CT image–training and the anatomical diversity of these vessels [10,35]. Therefore, to overcome these limitations, further work would require the generation of 3D CT images with larger and more diverse data sets. The second most obviously artificial feature was an abnormal contour of the movable organs. This could be another limitation in the GAN-based realistic image synthesis. Recently, more powerful GAN models have been introduced into the medical field. We believe that many problems raised here can serve as criteria to test the performance of the newly introduced GAN models.

As expected, learning artificial features in the synthetic images improved the performance of radiologists in identifying artificial images. However, it did not reach our expectations. This is because artificial features occurred mainly in some images of certain anatomical subgroups. In addition, as mentioned before, it is not easy for radiologists to distinguish these artificial features from motion artifacts or noise in real images. Furthermore, our visual Turing tests were based on reviewing 2D synthetic CT slices. However, although 3D data (eg, CT) are presented as 2D images, human perception of an anomaly is based on the imagination of space from 2D images. These factors could make it difficult to determine whether each CT image is real or artificial.

### Comparison With Prior Work

Bermudez et al [36] reported that GAN can successfully generate realistic brain MR images. However, unlike this study, the previous GAN-based unconditional synthesis of advanced radiological images (CT or magnetic resonance images) has been confined to some specific pathologic lesions (eg, lung and liver lesions) and specific organs (eg, heart and brain) for a variety of purposes [8,36-40]. In contrast, this study shows that realistic high-resolution (512×512 pixels) whole-body CT images can be synthesized by GAN. GAN was trained with whole-body CT images (512×512 pixels) in this study; therefore, the model learned the semantics of the images. It is worth noting that the generated images cover a wide range of 2-dimensional (2D) slice CT images along the z-axis from the thorax to the pelvis and contain multiple organs. To the best of our knowledge, there has been no study that has investigated and validated the unsupervised synthesis of highly realistic body CT images by using a PGGAN.

### Limitations

Our study had some limitations. First, technical novelty is lacking in this study. However, while state-of-the-art GAN models such as PGGAN and StyleGAN were introduced recently, there are still limited studies in the medical domain and a lack of published studies on anomaly detection tasks. As far as we know, this is the first attempt to generate high-quality medical images (whole-body CT) and to validate the generated medical images by expert radiologists. This study will provide readers a way to follow our approach and to achieve advances in anomaly detection tasks in medical imaging. Second, our training data are not enough to cover the probability distribution of normal data. This preliminary study used normal CT images from our institution. The training data consisted of relatively homogeneous CT images with similar acquisition parameters and CT machines. Therefore, further studies should focus on the collection of multi-center and multi-country diverse CT data to achieve better results. Third, due to limited graphics processing unit memory, our study only validated the realistic nature of separate 2D high-resolution body CT slices that were randomly generated by the GAN. This study did not handle 3D synthetic CT images, although real body CT images are volumetric data. Therefore, interslice continuity of pathologic lesions and organs may be a crucial factor for improving the performance of deep learning–based models. Further studies are needed to generate and validate 2.5D or 3D synthetic CT images in terms of detailed anatomical structures. Fourth, the number of synthetic images in the validation set varied between each anatomical region; thus, the statistical power may have been insufficient. However, we tried to avoid any researcher-associated selection bias in this process. Finally, we did not evaluate the correlation between the number of CT images in the training set and the generation of realistic images in the validation set. Our study showed that the PGGAN can successfully produce realistic body CT images by using a much smaller amount of training data in contrast to previous studies on the generation of celebrity face images with 1K pixels by 1K pixels [28,29]. However, we did not provide a cut-off value for the number of CT images required to generate realistic images. Therefore, further studies are needed to clarify the approximate data set size required for the generation of highly realistic normal or disease-state CT images.

### Conclusions

GAN can synthesize highly realistic high-resolution body CT images indistinguishable from real images; however, it has limitations in generating body images in the thoracoabdominal junction and lacks accuracy in anatomical details.

## Appendix

Multimedia Appendix 1 Example video of the multi-level random generation of synthetic chest computed tomography-lung window by the progressive growing generative adversarial network.

Multimedia Appendix 2 Example video of the multi-level random generation of synthetic chest computed tomography-mediastinal window by the progressive growing generative adversarial network.

Multimedia Appendix 3 Example video of the multi-level random generation of synthetic abdominopelvic computed tomography images by the progressive growing generative adversarial network.

Multimedia Appendix 4 Subgroup analysis of diagnostic performance with respect to the anatomical subgroups. A. Accuracy, B. Sensitivity, C. Specificity. There was a significant difference in accuracy (*) and specificity (†) between the thoracoabdominal junction (TA) and other image subgroups. Chest-L: chest computed tomography-lung window; chest-M: chest computed tomography-mediastinal window; AP-CT: abdominopelvic computed tomography.

## Abbreviations

AP-CT: abdominopelvic computed tomography
Chest-L: chest computed tomography-lung window
Chest-M: chest computed tomography-mediastinal window
CT: computed tomography
GAN: generative adversarial network
PGGAN: progressive growing generative adversarial network
