# Supplementary figures and images for: Realistic High-Resolution Body Computed Tomography Image Synthesis by Using Progressive Growing Generative Adversarial Network: Visual Turing Test
Source: JMIR Med Inform. 2021 Mar 17;9(3):e23328. doi: 10.2196/23328 (PMC8077702; doi:10.2196/23328)

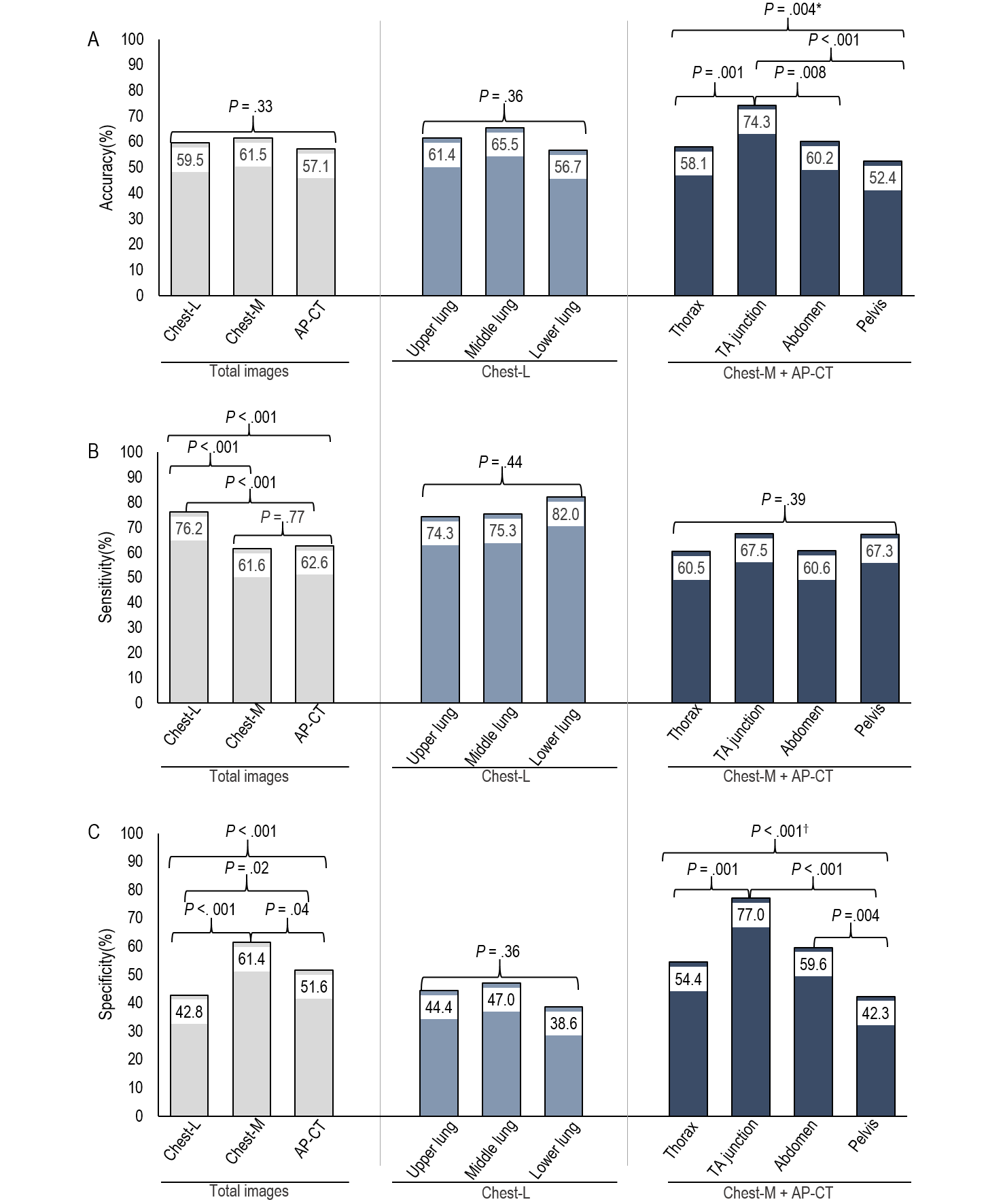

Supplement: Multimedia Appendix 4 [file medinform_v9i3e23328_app4.png]
